# Opportunities and Challenges for Next-Generation Thick Cathodes in Lithium-Ion Batteries

**DOI:** 10.3390/ma18153464

**Published:** 2025-07-24

**Authors:** Shengkai Li, Yuxuan Luo, Kangchen Wang, Lihan Zhang, Pengfei Yan, Manling Sui

**Affiliations:** 1 State Key Laboratory of Materials Low-Carbon Recycling, Beijing University of Technology, Beijing 100124, China; klyn_lee@outlook.com (S.L.); pfyan@bjut.edu.cn (P.Y.); mlsui@bjut.edu.cn (M.S.); 2 Beijing Key Laboratory of Microstructure and Property of Solids, Faculty of Materials and Manufacturing, Beijing University of Technology, Beijing 100124, China; yuxuanluo@emails.bjut.edu.cn (Y.L.); wkc852963@163.com (K.W.); 3 Institute of Matter Science, Beijing University of Technology, Beijing 100124, China

**Keywords:** lithium-ion batteries, thick cathode, architecture engineering, pore engineering, high energy density

## Abstract

Advancements in structural engineering are expected to enhance the wide-range commercial application of lithium-ion batteries by enabling the implementation of thicker cathode materials. Increasing the thickness of these cathodes can yield significant increasements in gravimetric energy density while concurrently lowering manufacturing costs. These improvements are pivotal to the successful commercial deployment of sustainable transport systems. However, several substantial barriers persist in the adoption of such microstructures, including performance degradation, manufacturing complexities, and scalability concerns, all of which remain open areas of investigation. This review delves into the obstacles associated with current modifying techniques in thick cathodes and explores the potential opportunities to develop more robust and thicker cathodes, while ensuring long-term performance and scalability. Finally, we provide suggestions on the future directions of thick cathodes to promote their large-scale application.

## 1. Introduction

Lithium-ion batteries (LIBs) have become the core of modern energy storage systems, owing to their wide range of applications spanning from portable electronic devices to electric vehicles (EVs) [1,2,3,4]. In 2019, the Nobel Committee honored John B. Goodenough, M. Stanley Whittingham, and Akira Yoshino with the Chemistry Prize in recognition of their groundbreaking work and significant advancements in LIBs. However, the continuous advancement of LIBs’ technology has become crucial with the increasing demand for higher energy density and longer cycle life for EVs in both industrial and academic communities [5]. One of the most important limiting factors lies in the cathode side which determines the energy density and occupies ≈ 40% of total manufacturing cost in LIBs [2].

Thick cathode design is an effective approach to improve the energy density of LIBs and reduce capital by increasing the load of active materials and reducing the proportion of non-active components such as conductive agents and binders simultaneously. Although the thick cathode has great potential, its practical application still faces many challenges including performance degradation, manufacturing complexity, and the feasibility of large-scale production [6,7]. For example, the energy density of the nickel-rich-cathode (i.e., a commercial cathode material with high specific capacity)-based LIBs is only about 250 Wh/kg, which is far behind the energy density demand target of the automobile industry (i.e., 400~500 Wh/kg) at present [8]. Thus, understanding the opportunities and challenges for next-generation thick cathodes in LIBs is important.

Remarkable progress in research and development of thick cathodes in LIBs has been made in recent years [9,10,11,12,13,14,15,16,17,18,19,20,21]. In terms of battery performance, few reviews summarized that thick cathodes have problems of poor rate performance and insufficient cycle stability due to the limited lithium ion diffusion, complex electron conduction paths, and increased interface impedance [9,10,11,12,13,14]. Yu and co-workers summarized corresponding gradient thick cathode design for high-energy and high-power LIBs [15], while Du et al. focused on limiting factors in thick cathode performance as applied to high-energy-density LIBs [21]. In addition, in terms of the manufacturing process, numerous reviews summarized that the traditional thick cathode coating process is prone to cause problems including coating cracking formation and particle breakage [18,19,21]. In addition, Fleetwood et al. demonstrated that the uneven distribution of conductive agent and binder will aggravate the interface resistance and further affect the cathode performance [16].The last ten years have witnessed substantial progress and significant understandings of breaking through the dynamic bottleneck of thick cathode by hole engineering, conductive network optimization, and innovative manufacturing process [22,23,24,25,26,27,28]. However, a comprehensive and updated review especially on both performance- and manufacture-related challenges on thick cathodes is highly desirable for readers to gain an overview picture of these two kinds of important challenges and corresponding optimization strategies on thick cathodes.

In this review, we aim to systematically analyze the performance and manufacturing challenges faced by thick cathodes in LIBs and explore their future optimization strategies. Through an in-depth study of key technologies such as pore engineering, innovative cathode architecture design (e.g., 2D conductive network and 3D skeleton structure) and electrode/electrolyte interfacial engineering, the breakthrough of thick cathodes is systematically summarized with high energy density, high power density and large-scale utilization potentiality, which lays a solid foundation for their wide application in EVs. Finally, the performance bottlenecks, manufacturing problems and corresponding future modifications of thick cathodes will be discussed in detail to promote their future large-scale applications.

## 2. Challenges for Thick Cathodes

Thick cathodes provide the possibility of an efficient improvement in the energy density of lithium-ion batteries (LIBs). These improvements are obtained by increasing the amount of active cathode materials while removing potential inactive components (e.g., binders and conductive agents). However, the implementation of thick cathodes remains a significant barrier, including performance-related challenges (e.g., poor power density characteristics and limited ion transport) and manufacturing difficulties at scale by using the present process. These two aspects will be discussed in detail below.

### 2.1. Performance-Related Challenges of Thick Cathodes

**Figure 1:**
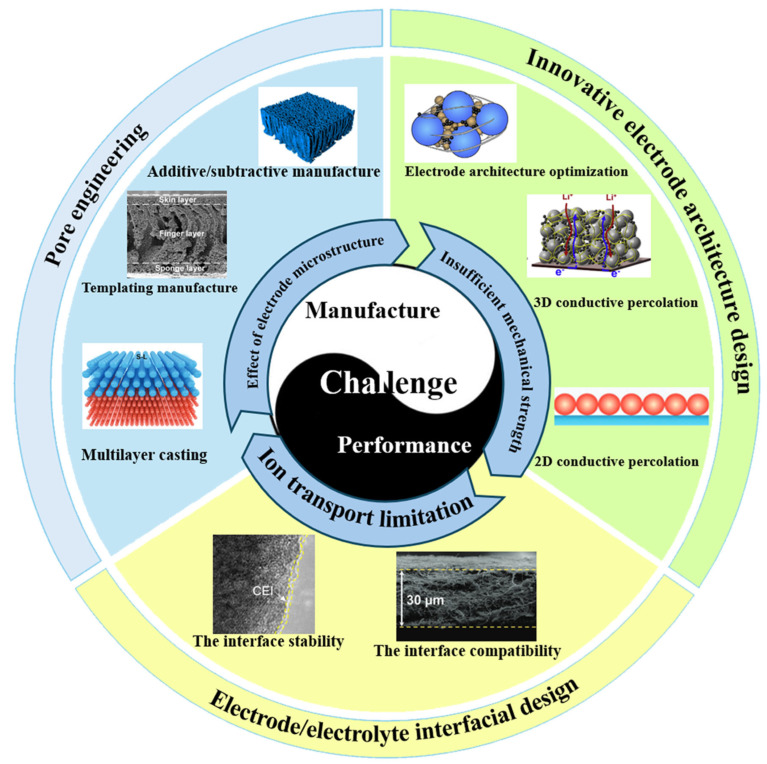
Schematic diagram of the relationship between manufacturing challenges and performance optimization strategies in thick cathodes design [33,34,35,36,37,38,39,40].

Thick cathode has broad application prospects in high-energy-density LIBs, but their performance and manufacturing challenges cannot be ignored (Figure 1). Thick cathode is regarded as the key technology to improve the energy density of LIBs due to its higher active material loading. However, there are some problems such as poor rate performance and insufficient cycle stability of thick cathodes. The research difficulty lies in how to improve the electrochemical performance of thick cathodes by optimizing cathode design parameters, such as cathode thickness, porosity, tortuosity, channel width, and spacing. Increasing the thickness of the cathode and the loading of the active material is one of the most direct means to increase the energy density. However, as the cathode thickness increases, the energy density of the LIBs demonstrates an initial rapid growth followed by a negligible increase [18]. When the cathode thickness exceeds a critical threshold, further increases in cathode thickness can result in the elongation of the electrolyte transport path. This elongation can inevitably increase internal resistance, reduce the efficiency of ion transport and consequently limit the final achievable energy density [21,29]. Achieving the optimal active material loading range for the highest energy density is one of the key challenges for thick cathodes, and the limitations of blindly pursuing ultra-thick cathodes should be avoided. The increase in the thickness will also be accompanied by a series of problems such as high tortuosity, slow transfer of Li^+^/Na^+^ transport, improper porosity, cracking, etc., which are the main reasons for the rate limit [30,31,32]. Due to the directionality of particle arrangement and high compaction density, thick cathodes often form a pore structure with high tortuosity. High tortuosity leads to limited lithium ion diffusion and decreased rate performance, and more obvious capacity degradation at high rates [19].

Insufficient porosities affect the continuous ion flow channel, contributing to increased cathode tortuosity and resulting in the poor electrolyte wettability. However, the low rate performance can be optimized by reducing the electronic resistance. The final result is a sharp decrease in the ionic conductivity of the entire cathode, resulting in a significant increase in the ion overpotential and a rapid decrease in capacity during charging/discharging [20]. Excessive porosity provides sufficient electrolyte infiltration, but the electron conduction path is dispersed. By integrating carbon nanotube networks, the electron conduction path can be improved, and thus the rate performance can be significantly improved [22]. Designing a double-layer cathode with higher porosity near the separator increases the electrolyte permeation rate compared to a uniform cathode [23,24]. For example, Xu et al. designed a bimodal microscale porous structure [25], where the synergistic effect of different pore sizes effectively reduces ion transport resistance: the large pores serve as electrolyte storage, and the small pores provide ion transport channels. These examples illustrate the potential for using pore engineering to accurately balance porosity, thereby improving thick cathode performance.

The performance of thick cathodes is inextricably related to cathode microstructure architecture. The optimization of microstructure is the key to improving the energy density, power density, cycle stability, and safety of thick cathodes.

### 2.2. Manufacture-Related Challenges of Thick Cathodes

**Figure 2:**
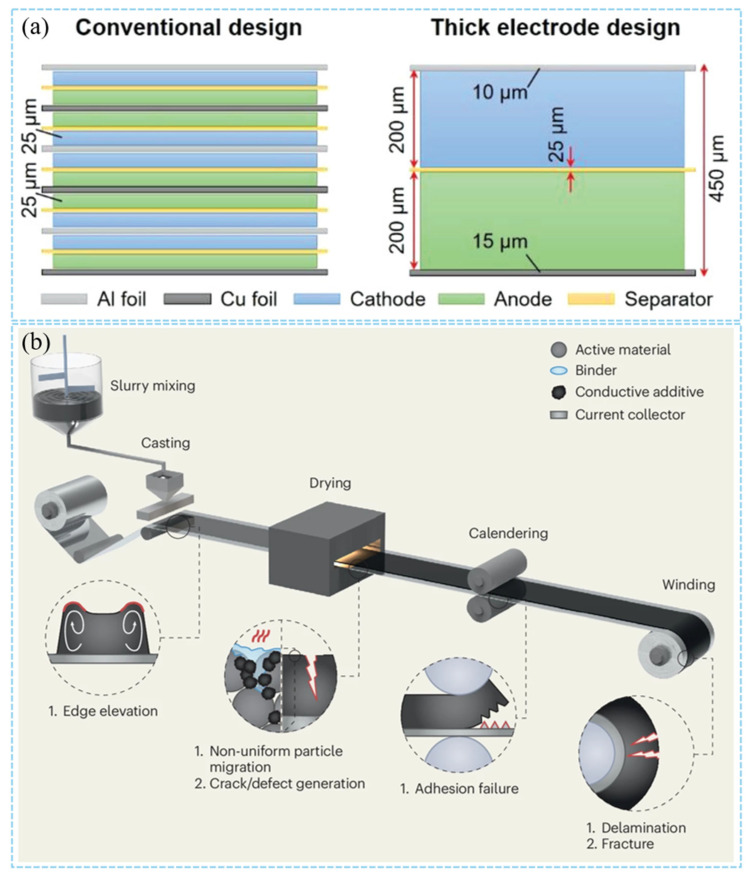
(**a**) The comparison diagram of thin electrode stacking structure and thick electrode structure [12]. Reproduced with permission from [John Wiley and Sons], [2019]. (**b**) Schematic diagram of cathode preparation process [7]. Reproduced with permission from [Springer Nature], [2025].

Increasing the thickness of the cathode can significantly enhance the loading of active materials. This thick cathode design effectively reduces the proportion of non-active components, such as current collectors and separators, within the battery (Figure 2a). Furthermore, this approach not only lowers the manufacturing cost of the battery but also improves its volumetric energy density. As a key technology to improve the energy density of LIBs, the design of thick cathodes faces multiple complex challenges in the manufacturing process. The heterogeneity of cathode structure is the primary problem. The non-uniform distribution of conductive additives (such as carbon black) and binder will lead to the obstruction of the electron conduction path. This inhomogeneity will aggravate the interface resistance and reduce the utilization rate of active materials especially in thick cathodes. Studies have shown that the uniformity of conductive additive and binder domain distribution can be improved by regulating the migration behavior of binders (such as optimizing drying conditions or using new binder systems), thereby improving the overall performance of the cathode [26]. In addition, the high load characteristics of thick cathodes can easily lead to mechanical failure. The capillary stress caused by solvent evaporation during drying can lead to coating cracking, while the plastic deformation during compaction may destroy the contact between particles. In response to these problems, researchers have proposed a variety of strategies: the introduction of water/isopropanol mixed system can reduce the capillary pressure during the drying process and inhibit crack formation [27]; laser drilling or 3D current collector design can alleviate the bottleneck of ion transport [28]. The dry cathode process significantly improves the structural integrity of the thick cathode by avoiding the solvent volatilization problem of traditional coating [41]. In terms of electrochemical performance optimization, single-crystal cathode materials (such as LiNi_0.8_Mn_0.1_Co_0.1_O_2_) have become an ideal choice for thick cathode design due to their excellent mechanical stability and low volume expansion characteristics [42]. Combined with optimized electrolyte formulations (such as those containing LiBO_2_ additives [43]), the interface side reactions can be further suppressed.

The cathode manufacturing process of LIBs is a complex process, which is mainly completed by four steps: mixing–coating–drying–calendering (Figure 2b). Each stage of the cathode manufacturing process has a decisive influence on the final cathode structure and the resulting electrochemical performance [44,45]. The degree of calendering, the type of conductive agent, and the different cathode manufacturing processes have a strong impact on the final electrochemical performance of the cathode [46]. Nowadays, the traditional cathode manufacturing process has become relatively more perfect. However, there are still considerable challenges for the improvement of the thick cathode manufacturing process due to the limitations of thick cathode thickness, porosity, ion conduction velocity, and other factors.

In the mixing step, the mixtures commonly used in the preparation of cathodes include active materials, carbon black, binder, and solvents that adjust the viscosity of the slurry. Lee et al. prepared thick cathodes with controllable crack density by adjusting the solid content in the slurry, solvent volatility (N-methyl-2-pyrrolidone, N-Dimethylformamide, acetone), and binder distribution [47]. Further exploration of the slurry formulation and the mixing process has a very important impact on improving cathode performance. In the manufacturing process of thick cathodes, the most obvious problem is that the surface of the cathode is cracked due to the excessive thickness of the cathode. The capillary stress generated by the traditional coating process after drying or calendering during the preparation of thick pole pieces may cause pole pieces to crack. Optimizing the structural stability of cathode materials is critical, especially for their high-capacity cathode materials with large volume expansion.

In the coating-drying step, different process parameters (such as blade gap, coating speed, drying temperature, and air flow rate, etc.) have different effects on the physical properties (e.g., thickness, surface load, and porosity) and electrochemical properties (capacity and rate performance) of the cathode. The coating weight and porosity are the key factors affecting the performance of the cathode [48].

Finally, the microscopic pore structure of the coating was fixed by the calendering process to determine the electrochemical performance of the cathode, and then the cathode was assembled into a variety of battery configurations. Calendering is a key cathode process step in the manufacture of LIBs, which directly affects the volume energy density and mechanical properties of the cathode [49]. The coating is compacted to the desired porosity by calendering, and the pore structure of the cathode coating is very important for electrochemical performance.

Therefore, in order to improve industrially compatible thick cathodes, we need to pay attention to key challenges such as pore engineering, innovative cathode architecture design, electrode/electrolyte interfacial design, and corresponding strategies, which will be discussed in the following chapters.

## 3. Strategies to Enhance Thick Cathodes’ Performances

Through the comprehensive application of pore engineering, innovative cathode architectures, electrode/electrolyte interface design, and other strategies, the performance of thick cathodes can be effectively improved, thereby promoting the development of LIBs. First, pore engineering is one of the important strategies to optimize ion transport and electron conduction by adjusting the porosity of the cathode. Increasing the porosity helps ion transport, but too high a porosity limits electron conduction and affects the wettability of the electrolyte. Therefore, finding the appropriate porosity is the key to improving the electrochemical performance of LIBs. This chapter also introduces three main pore engineering design strategies: additive/subtractive fabrication [50,51,52], ex situ/in situ templating fabrication [35,53,54], and multilayer casting procedure [34,55]. Secondly, innovative cathode structure design is also the key to improve the performance of thick cathodes. The 2D conductive percolation network-based current collector, such as graphene [56] and carbon nanotubes [57,58], provides efficient electron and ion transport channels. The construction of a 2D nanosheet structure by self-assembly technology can effectively optimize the ion transport path [59]. In addition, 3D conductive scaffold-based current collectors, such as metal foams [60,61] and carbon nanotube frameworks [62], provide low-resistance electron transport paths and ion diffusion channels, significantly improving the overall performance of the cathode. The dry cathode process eliminates the use of N-methyl-2-pyrrolidone (NMP) and conforms to the trend of green manufacturing [62,63,64]. The design of the electrode/electrolyte interface is also important. Optimizing the electrode/electrolyte interface behavior through interface engineering, inhibiting interface side reactions, and enhancing structural stability are effective strategies to improve the performance of thick cathodes. Choosing appropriate electrolyte materials and optimizing interface modification can significantly improve the cycle life and high rate performance of the battery.

### 3.1. Pore Engineering

In general, the cathode porosity of commercial LIBs is usually 20% to 40% [65]. The increase in porosity is beneficial to ion transfer, but too much porosity will limit electron conduction. Too low porosity may lead to insufficient electrolyte infiltration and increased interface impedance. Only an appropriate porosity can ensure the maximum electrochemical performance of the cathode. At present, the widely reported pore engineering design strategies can be divided into three categories: additive/subtractive manufacture, ex situ/in situ templating manufacture, and multilayer casting procedure.

#### 3.1.1. Additive/Subtractive Manufacture

The advantages of cathode pore engineering have been firmly established through both theoretical analyses [66] and experimental verifications [67]. A multitude of approaches of pore engineering in thick cathodes have been investigated, which can be predominantly categorized into two distinct types: additive/subtractive manufacturing and ex situ/in situ templating techniques. This classification provides a systematic framework for understanding and optimizing cathode architectures, enabling researchers to tailor pore structures precisely to enhance electrochemical performance.

**Figure 3:**
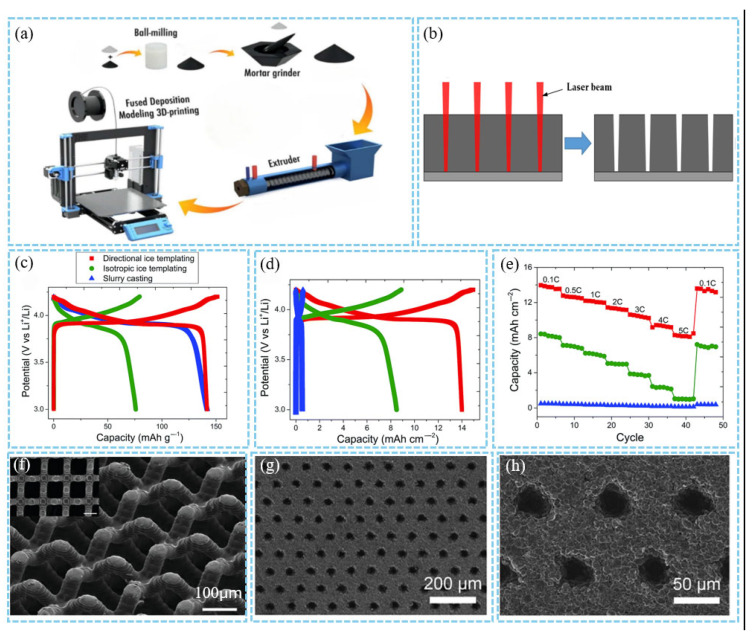
(**a**) Schematic of the 3D printing process [68]. (**b**) Schematic representation of laser structuring strategies [69]. Charge and discharge profiles at 0.1 C for the LCO electrodes, showing (**c**) gravimetric capacities [54] and (**d**) corresponding areal capacities [54]. (**e**) Reversible areal capacities of the three types of electrodes at different current rates. Scanning electron microscopy (SEM) images [54]. Reproduced with permission from [Royal Society of Chemistry], [2013]. (**f**) C-based anode made by 3D printing [51]. Reproduced with permission from [John Wiley and Sons], [2020]. (**g**,**h**) C-based anode made by laser patterning [70]. Reproduced with permission from [Elsevier], [2020].

Additive manufacturing methods include coextrusion, which involves lab-synthesized rods mixed with an LCO-polymeric binder and a carbon axial core. This process is followed by pyrolysis [52]. Additionally, as shown in Figure 3a, C-based anodes are fabricated via 3D printing, UV curing, and pyrolysis of acrylate-based resins [68]. Another method, ice templating, fabricates porous materials by controlling the directional solidification of ice in a suspension, with pore structures shaped through ice crystal growth and sublimation [54].

These methods can significantly enhance energy density, like the ice-templating method. Figure 3c shows the galvanostatic (dis)charge profiles. The 900 μm-thick DIT electrode exhibits a reversible gravimetric capacity of 142 mA h/g at 0.1 C, comparable to the much thinner (26 μm) SC electrode, while the 900 μm-thick IIT electrode only reaches a specific capacity of 76 mA h/g. This breaks the common view that thick cathodes have low capacity. In terms of areal capacity (Figure 3d), the DIT electrode achieves 14 mA h/cm^2^ at 0.1 C, surpassing IIT (8.4 mA h/cm^2^) and SC (0.5 mA h/cm^2^). At 5 C, the volumetric capacity of DIT is 92 mA h/cm^3^, far higher than that of IIT (11 mA h/cm^3^) and SC (53 mA h/cm^3^), demonstrating the thick and open-structured DIT’s superiority in high rate volumetric capacity. For rate capability (Figure 3e), the capacities of SC and IIT electrodes drop by 72% and 88%, respectively, when the rate increases from 0.1 C to 5 C, whereas that of DIT only decreases by 41%. The oriented micropores in DIT maintain efficient Li^+^ transport, breaking the high rate capacity fading limitation of disordered/traditional structures [54].

One of the main advantages of additive manufacturing is its highly ordered manufactured microstructure (Figure 3f), low tortuosity, and high rate performance [51]. However, a drawback is that the pore channel diameters tend to be large due to the resolution limitations of the processing equipment [53]. Subtractive manufacturing methods include femtosecond laser sintering (Figure 3b). As shown in Figure 3g,h, this technique uses an ultrashort pulse laser as the power source to sinter powdered materials in a low-oxygen environment [69]. A key benefit of this approach is that the cathode-casting process does not require significant modification. Additionally, it ensures high compatibility with existing manufacturing techniques [70,71]. Nonetheless, the drawbacks of subtractive manufacturing techniques involve comparatively elevated capital expenditures and extended financial amortization periods [12], low throughput, longer production time, and the possibility of droplet generation within the laser cutting kerf resulting from partial melting of cathode materials [72].

#### 3.1.2. Ex Situ/In Situ Templating Manufacture

From a manufacturing perspective, we give a concise overview of previous studies on template manufacturing in pore engineering and categorize them into two groups: ex situ templating manufacture and in situ templating manufacture. In experiments aimed at increasing cathode thickness to achieve the volumetric energy density at the cell and battery pack levels, the advantages of these template-based pore engineering routes for thick cathodes have been fully demonstrated [10].

In materials synthesis, the ex situ templating strategy encompasses the fabrication of a sacrificial template (e.g., porous polymers, and nanoparticles), followed by infiltration or deposition of the target phase, and subsequent template removal via thermal decomposition or chemical etching to yield hierarchically structured porous materials. In situ templating leverages dynamic self-assembly, sol–gel chemistry, or phase separation mechanisms. These mechanisms generate templates concurrently with the formation of the matrix material. This approach obviates the need for prefabricated templates. Additionally, it enables the direct formation of mesoporous or ordered nanostructures.

**Figure 4:**
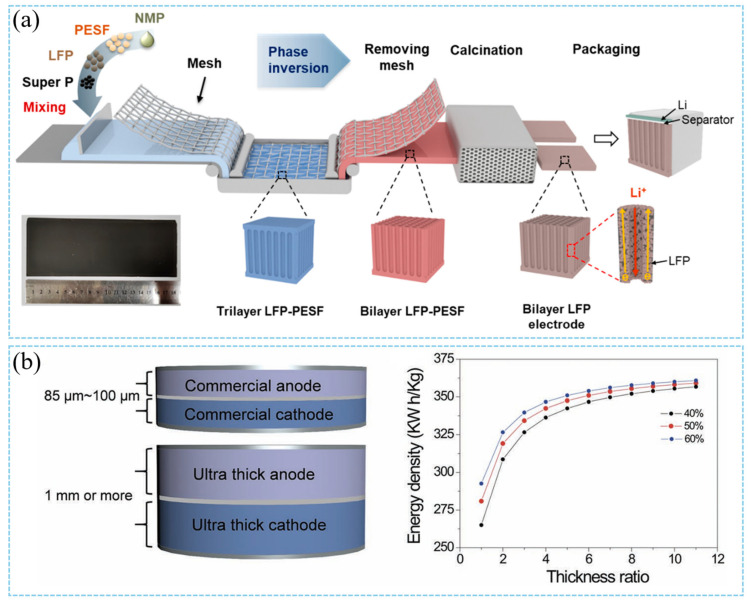
(**a**) Figure depicting the preparation process of a double-layer lithium iron phosphate (LFP) electrode via the templated phase inversion technique [35]. Reproduced with permission from [American Chemical Society], [2021]. (**b**) The plot illustrates energy density trends of LCO-graphite cells as electrode thickness increases under varying porosities [74]. Reproduced with permission from [John Wiley and Sons], [2018].

**Figure 5:**
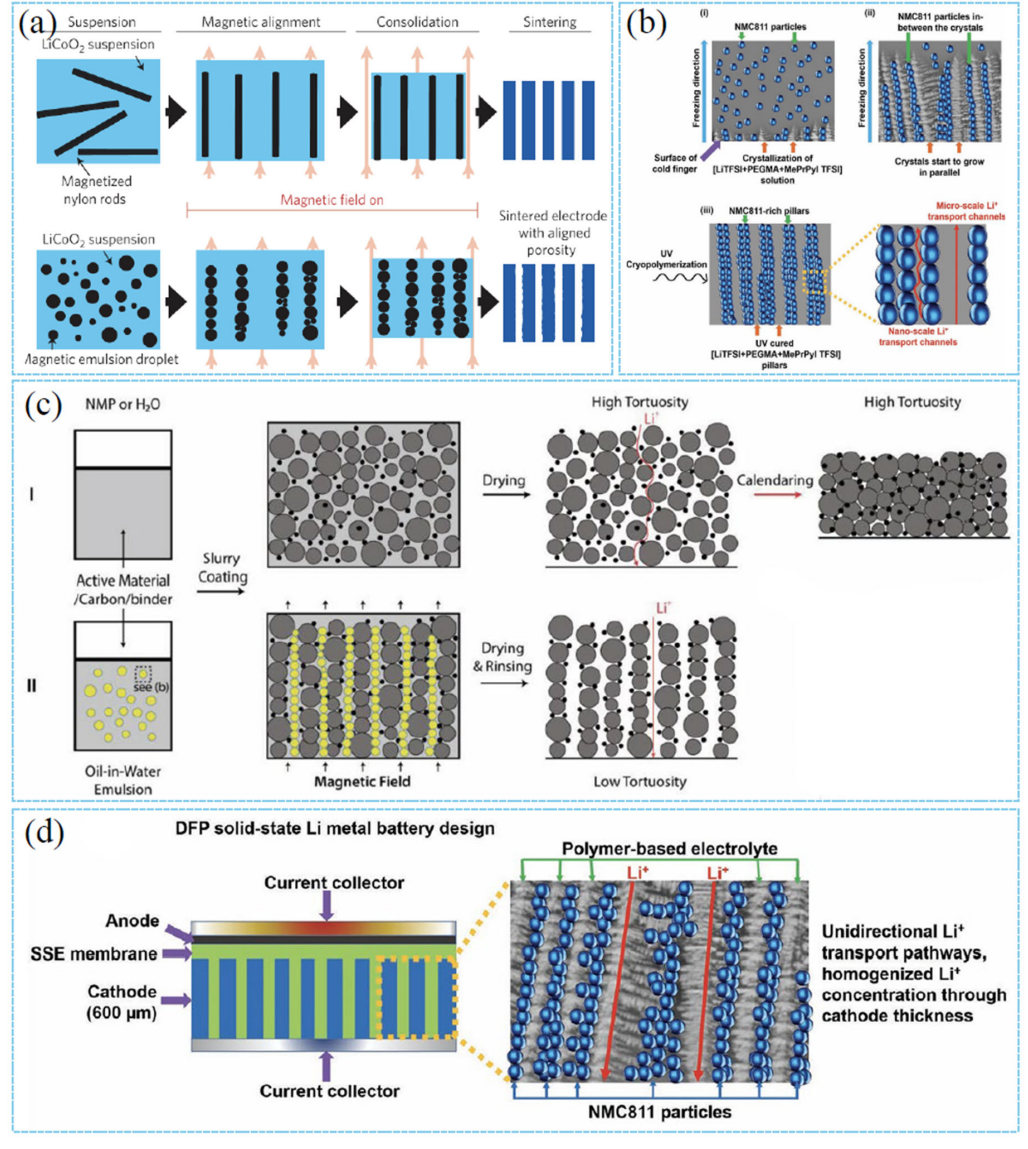
(**a**) Fabrication of electrodes via magnetic alignment of magnetic microrods embedded in a sacrificial phase [53]. Reproduced with permission from [Springer Nature], [2016]. (**b**) Schematic illustration of the preparation of low-tortuosity electrodes in comparison with that of conventional electrodes [76]. (**c**) The SSLMB architecture features an anisotropic cathode structure featuring vertically oriented NMC811-enriched pillars enclosed by a polymer-based electrolyte [75]. Reproduced with permission from [John Wiley and Sons], [2018]. (**d**) The procedures involved in the directional freezing-polymerization (DFP) technique for fabricating anisotropic cathode architectures [76].

In recent studies, innovative cathode fabrication methods such as templating have been proposed in the laboratory. The cracking and ion transport issues of thick cathodes are mitigated by constructing special microstructures (such as porous networks). The templated phase inversion method, pioneered by Wu and co-workers, conducts a low-tortuosity LiFePO4 (LFP) cathode with ultra-high loadings of active materials and a highly efficient transport network [35]. This can be classified as a specific implementation of ex situ templating manufacture. The low-tortuosity and mechanically robust ultra-thick cathodes constructed via the template phase inversion method exhibited aligned open-pore structures with low tortuosity, which can significantly improve charge transport kinetics and increase accessible active sites in thick cathodes. The aligned low-tortuosity pores along the cathode depth direction reduce the Li^+^ diffusion distance and facilitate Li^+^ diffusion within the pores (Figure 4a). This technology constructs microchannel structures using stainless steel mesh templates, involving independent template (network) preparation in the early stage, followed by structure formation through contact with the slurry, and subsequent template removal in the later stage [35].To address the challenge of limited lithium-ion transport, Chun Huang’s research group has employed a scalable ice templating technique to fabricate 900-μm-thick cathodes featuring aligned pore arrays along the dominant ion transport direction, eliminating the need for post-processing sintering [54]. Additionally, freeze-casting technology has been employed to fabricate LiNi_0_._8_Co_0_._15_Al_0_._05_O_2_ (NCA) cathodes with controllable oriented pores. The cathode tortuosity was characterized via X-ray tomography combined with thermal diffusion simulation and electrochemical transport measurements [73]. Taking inspiration from the vertical microchannels in natural wood, which act as efficient highways for water transportation, researchers have designed advanced water transport materials. The microstructural architecture of wood has been successfully replicated into ultra-thick bulk LiCoO_2_ (LCO) cathode through a sol–gel process [74]. This strategy enables the realization of high areal capacity and superior rate capability. The ultra-thick LCO cathodes fabricated via natural wood templating technology inherited a characteristic microstructure with low tortuosity, which significantly facilitates lithium-ion transport within the cathode. Notably, this approach allows for the fabrication of LCO cathodes with a thickness of up to 1 mm, approximately 12 times the thickness of commercial LCO electrodes (Figure 4b). Alternatively, innovative designs such as thick cathodes with low tortuosity represent another promising pathway to achieve higher energy density and lower costs [53,75]. J.S. Sander’s team has demonstrated that magnetically controlled sacrificial features can create oriented pore arrays in lithium-ion cathodes [53]. These oriented pores enable faster charge transport kinetics and endow the cathodes with more than triple the areal capacity at practical charge–discharge rates. The value of low tortuosity is evidenced by recent results on low-tortuosity lithium cobalt oxide (LCO) cathodes fabricated via two methods: co-extrusion with sacrificial porogens (carbon black) and directional freezing of aqueous suspensions. In one scenario, the sacrificial magnetic phase is composed of magnetically decorated microbars, whereas in the other scenario, it is made up of emulsified droplets of ferrofluids. Even though both methods are effective, their alignment mechanisms are fundamentally different (Figure 5a) [53]. Meanwhile, the emulsion-based magnetic alignment method can produce thick cathodes (with a thickness of >400 μm) featuring ultrahigh areal capacity (up to ≈ 14 mA h/cm^2^, compared to 2~4 mA h/cm^2^ for conventional LIBs). Conducted entirely at room temperature, this approach is characterized by simplicity and scalability, yielding LCO electrodes with a low tortuosity of 1.93 ± 0.03 (Figure 5b) [75]. The applications of these methods are also gradually expanding, as directional freezing and polymerization can be used to fabricate thick cathodes containing vertically interlaced cathode arrays and solid-state electrolyte materials for solid-state batteries [76]. As shown in Figure 5c, Chun Huang’s team fabricated a 600-μm-thick cathode composed of vertically aligned NMC811-rich pillars surrounded by an ionomeric electrolyte without the need for templates [76]. These hybrid cathodes were created via an innovative directional freezing and polymerization (DFP) process. In this process, active cathode particles and ionomers directly self-assemble into a preferentially anisotropic dense cathode structure without any subsequent pressing, heating, solid-state electrolyte (SSE) infiltration, or template removal steps (Figure 5d). This work represents one of the earliest reports on template-free vertically aligned cathode structures, exhibiting fast Li^+^ transport kinetics in solid-state lithium-metal batteries (SSLMBs) [76]. The DFP method should apply to various cathode materials.

#### 3.1.3. Multilayer Casting Procedure

**Figure 6:**
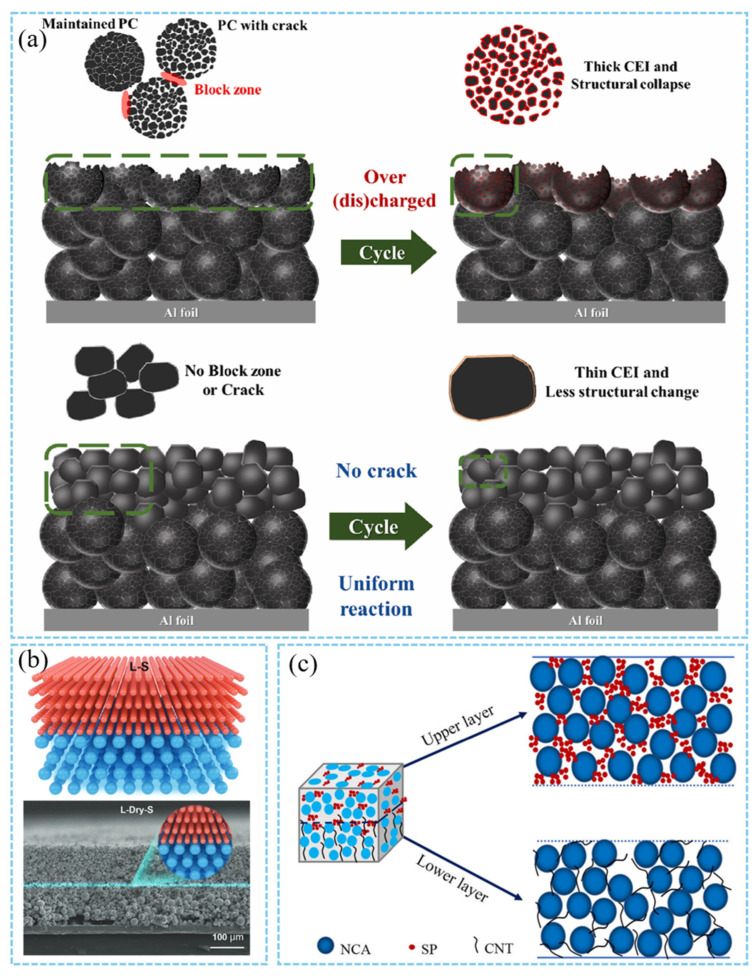
(**a**) Schematic illustration of the reason for the deteriorated performance of only the polycrystalline cathode (OP), the enhanced performance of the double layer cathode of poly (lower) and single (upper) crystalline (DB) [79]. (**b**) Schematic illustration of the particle-size double-layer architecture cathode [34]. Reproduced with permission from [John Wiley and Sons], [2024]. (**c**) Schematic diagrams of the dual-layered CNT-P cathode [85]. Reproduced with permission from [American Chemical Society], [2022].

Thick cathodes can significantly increase the energy density, but their limited ion/electron transport, large interface impedance, and poor cycle stability seriously restrict their performance. The traditional “homogeneous cathode” design is difficult to meet the needs of thick cathodes, and the gradient structure design provides a new idea for solving the above contradictions by regulating the spatial distribution of porosity, composition, or conductive network. Moreover, the multi-layer structure can significantly improve the adhesion force between the cathode and the current collector. Specifically, the adhesion strength of the multilayer cathode is 45% higher than that of the single layer. Additionally, this structure reduces the interface stripping problem during cycling [77]. Zhang et al. proposed a cathode structure with a gradient of pore size in the vertical direction: a small pore size (20–40 μm) near the diaphragm side and a large pore size (60–120 μm) near the current collector side [78]. Through this design, the ion transport channel near the diaphragm is expanded, the lithium ion diffusion distance is shortened, and the high active material loading of the small holes is leveraged to enhance capacity utilization. High-nickel NCM cathode materials (with Ni content ≥ 0.8) exhibit a high theoretical specific capacity (~280 mA h/g). However, they suffer from inferior mechanical robustness and suboptimal thermal stability [55]. Moreover, the thick cathode with high loading is prone to break the upper particles due to rolling pressure, resulting in uneven reaction, blocked lithium ion diffusion, and intensified interfacial side reactions. The single-crystal/polycrystalline layered structure design strategy is applied to thick cathode design while considering both mechanical strength and electrochemical performance [79]. The upper layer adopts single-crystal particles with high mechanical strength to avoid particle breakage caused by rolling pressure. The lower layer adopts polycrystalline particles to provide a rich active surface area and ion diffusion channels. Through this structure, the carbon black/PVDF agglomeration (“block zone”) caused by the upper layer crushing is effectively prevented, and the problems of uneven electrolyte penetration and excessive growth of CEI layer are reduced, thereby reducing the interface impedance (Figure 6a). It solves the core problem that the upper particles are easy to break and that the reaction is not uniform during the rolling process of the traditional thick cathode [79,80]. The interface peeling and ion transport blockage caused by binder migration during the drying process of thick cathodes, as well as the traditional single-layer coating process, make it difficult to effectively regulate the cathode’s internal structure. Reducing the content of the top layer binder through the binder gradient design can alleviate the “blocking effect” (binder choke points), reduce the ion diffusion resistance, and significantly improve the electron conduction ability of the cathode [81,82]. The thick cathode exhibits poor performance due to the limitation of lithium-ion transport during high rate discharge. How to improve the rate performance of the cathode while maintaining high energy density is an important challenge. By adjusting the particle size gradient and the electrolyte diffusion channel, the large-particle active material is used on the side near the current collector to reduce the ion diffusion distance (Figure 6b). The use of small active particles on the side near the separator accelerates electrolyte penetration and reaction kinetics, which significantly improves the performance and cycle life of LIBs at high rates [34,83,84]. Song et al. prepared a combination structure of two typical conductive agents in different layers by the layered coating method [79]. The combination of carbon nanotubes as the bottom conductive agent and super P as the top conductive agent (Figure 6c) successfully reduced the thickness of the electrode/electrolyte interface layer and improved the coulomb efficiency and cycle life of the battery [85]. The core of the gradient structure is to optimize ion/electron transport pathways through regulating spatial distribution. This optimization effectively addresses ion transport bottlenecks and interface mismatches in thick cathodes and demonstrates significant performance improvement in liquid/solid batteries. In the future, it is necessary to further simplify the process, improve interface compatibility, and explore new material systems to promote the large-scale application of gradient cathodes in commercial batteries.

### 3.2. Innovative Cathode Architecture Design

Structural innovation plays a key role in the development of thick cathodes. By optimizing the structure of the cathode, it can not only improve the energy density and cycle life of the battery but also reduce the manufacturing cost and improve the environmental protection performance. In addition, the method of structural innovation also has certain universality and can be extended to other types of cathode materials and energy storage devices.

#### 3.2.1. Two-Dimensional Conductive Percolation Network-Based Current Collector

The 2D conductive network structure (such as graphene, transition metal oxide nanosheets, graphite foam, etc.) provides efficient electron/ion transport channels and significantly improves the electrochemical performance of thick cathodes due to their high specific surface area, excellent conductivity, and structural controllability.

**Figure 7:**
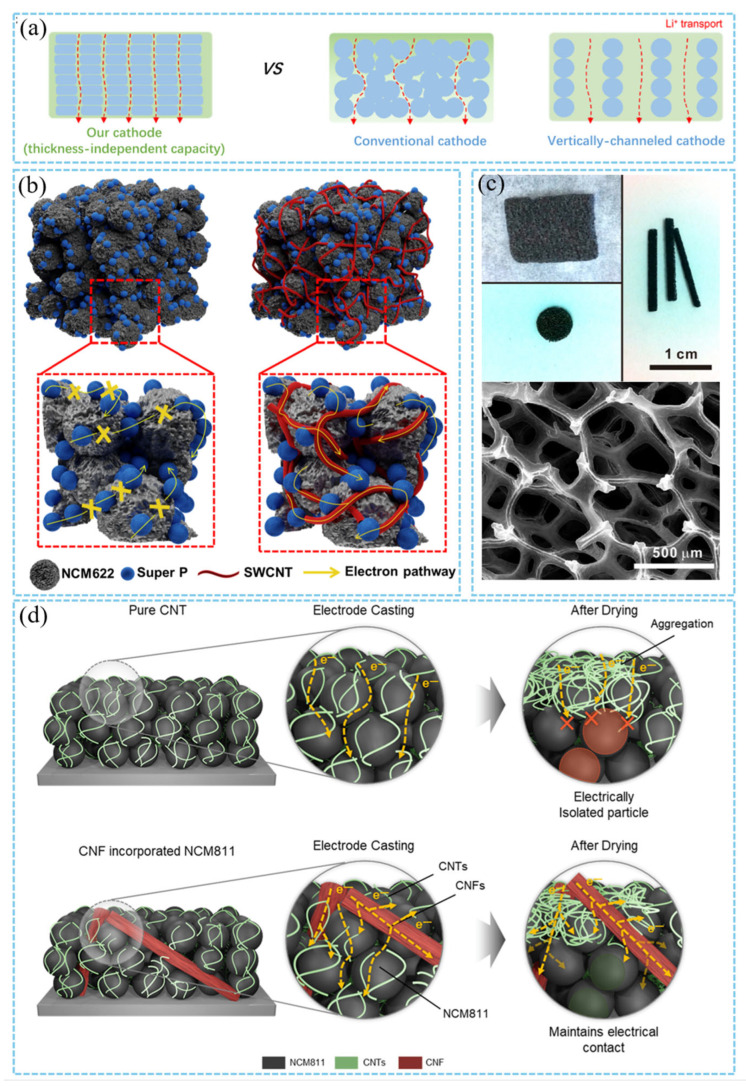
(**a**) Comparison diagram of 2D porous sheet and layered assembly structure [59]. Reproduced with permission from [Elsevier], [2021]. (**b**) Schematic representation of electronic transportation in the NCM622 cathode with Super P and SWCNT + Super P additives [57]. Reproduced with permission from [Elsevier], [2021]. (**c**) Photograph of UGFs in different geometries and SEM image of the UGF showing the microstructure of the UGF [56]. Reproduced with permission from [American Chemical Society], [2012]. (**d**) Schematic diagram shows that carbon nanofibers and carbon nanotubes build a strong electronic conduction network for high energy density NCM811 cathode materials [86]. Reproduced with permission from [American Chemical Society], [2024].

The construction of 2D nanosheet layered structure by self-assembly technology is a typical strategy for thick cathode design. For example, 2D porous NCM nanosheets provide fast lithium ion diffusion channels through in-plane pores (pore size 40 nm), while vertically aligned pores reduce ion transport distance (Figure 7a). The initial capacity of 147.2 mA h/g is still maintained in the thick cathode with a mass load of up to 320 mg/cm^2^, and the surface capacity is as high as 45.4 mA h/cm^2^, far exceeding the commercial cathode [59]. Traditional conductive agents (such as Super P carbon black) are easy to form isolated conductive networks under high loading, resulting in a decrease in the utilization of active substances. CNT is often used as the skeleton of a 2D conductive network due to its high aspect ratio and excellent conductivity. Woo et al. introduced 0.06 wt% SWCNT into the NCM622 cathode to form a continuous conductive network throughout the cathode (Figure 7b). Compared with the traditional conductive agent, SWCNT significantly reduced the charge transfer resistance (*R*_ct_ decreased from 34.1 Ω to 10.6 Ω), so that the cathode maintained 94% capacity after 50 cycles [57]. During the drying process of cathode fabrication, the migration of CNTs will lead to serious agglomeration of carbon additives on the surface and leave a poor conductive network in the whole cathode. Ali et al. solved this problem by optimizing the ratio of high-aspect-ratio carbon nanofibers (CNFs) to CNTs (0.25 wt% CNF + 0.75 wt% CNT) (Figure 7d) [86]. In addition, the flexible structure of SWCNT alleviates the volume expansion of the thick cathode during charging and discharging, and improves the mechanical stability. Similarly, the LiMn_2_O_4_@single-walled carbon nanotube (SWCNT) thick cathode was prepared by vacuum filtration combined with the freeze-drying method by Guo et al. The SWCNT was used as both a conductive agent and a binder to replace the traditional PVDF binder and acetylene black conductive agent, forming a conductive network on the surface of the nanosheets to support high-quality load (~190 mg/cm^2^) and maintain excellent rate performance [58].

The ion transport kinetics can be optimized by adjusting the porosity and surface morphology of 2D materials. Hallot et al. studied the effect of the ordered/disordered structure of LiMn_1.5_Ni_0.5_O_4_ films on the diffusion of lithium ions. The disordered spinel structure (Fd-3m) reduces the Li diffusion barrier due to the randomly distributed Ni/Mn sites, and its surface capacity still maintains an initial capacity of 84% at 10 C rate [87]. Ji et al. prepared ultra-thin graphite foam (UGF) by chemical vapor deposition. Its porous structure (pore size of 200–500 nm) provides a high specific surface area and low tortuosity. The unique 3D framework provides a continuous electron conduction path (Figure 7c). The active material is uniformly loaded on the graphite surface, which significantly reduces the interface contact resistance, so that the LFP cathode still outputs a specific capacity of 70 mA h g^−1^ at 1280 mA g^−1^ [56].

The 2D conductive network structure shows significant advantages in the design of thick cathodes by optimizing the ion/electron transport path and constructing a lightweight skeleton. Future research needs to focus on material porosity regulation, interface optimization, and large-scale preparation to promote its practical application in high-energy density batteries. With the advancement of technology and cost reduction, 2D conductive networks are expected to become the core design strategy for the next generation of high-performance LIBs.

#### 3.2.2. Three-Dimensional Conductive Scaffold-Based Current Collector

In recent years, as an innovative design strategy, the 3D conductive network structure has significantly improved the comprehensive performance of thick cathodes by constructing continues electron conduction channels and ion diffusion channels throughout the cathode thickness [37,60,61,62,88,89].

**Figure 8:**
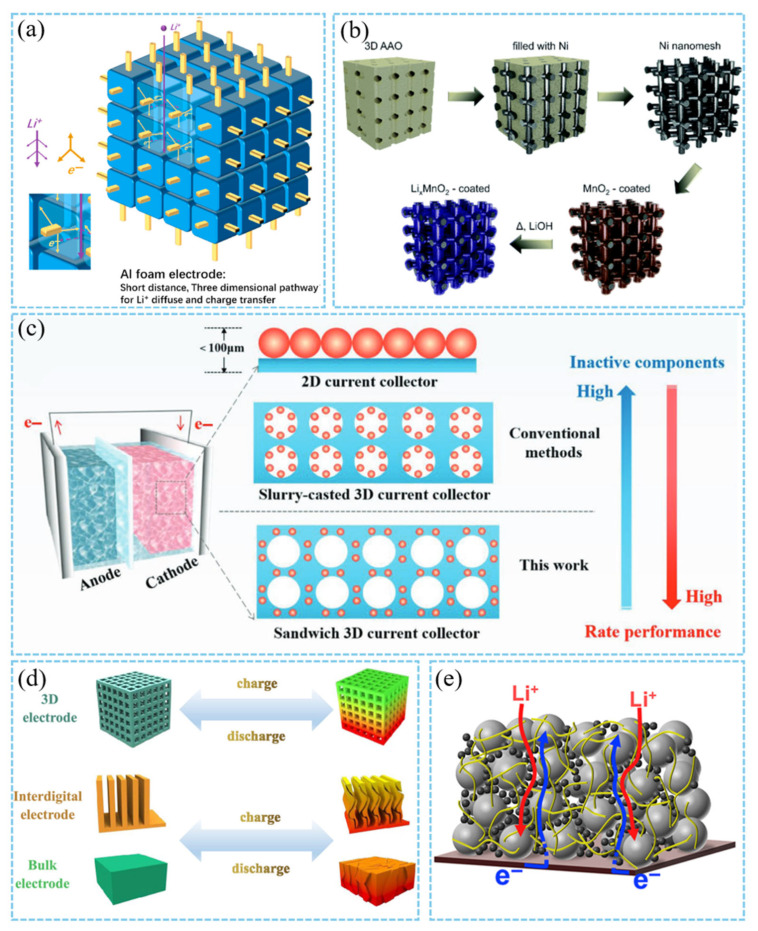
(**a**) The electron and lithium-ion transfer in the Al foam electrode [61]. Reproduced with permission from [Elsevier], [2022]. (**b**) Fabrication scheme of the nano-mesh cathodes [89]. Reproduced with permission from [Royal Society of Chemistry], [2013]. (**c**) Structural and performance differences in sandwich 3D current collectors and slurry-casted 3D current collectors [37]. Reproduced with permission from [John Wiley and Sons], [2019]. (**d**) Structural stabilities of the conventional bulk electrode, interdigital electrode, and 3D-printed electrode during the charging and discharging [92]. Reproduced with permission from [American Chemical Society], [2022]. (**e**) Schematic representation for electron/ion transport behavior of carbon fiber-interwoven cathodes [33]. Reproduced with permission from [Elsevier], [2016].

3D conductive networks (such as metal foams, carbon nanotubes, and graphene frameworks) provide low-resistance electron transport paths and reduce polarization by constructing continuous conductive frameworks [90]. Ni and Al foam current collectors having high specific surface area achieve efficient electron transfer in thick cathodes through a highly conductive skeleton (Figure 8a), although potential alloy corrosion issues such as Al surface passivation must be considered [60,61,91]. The 3D-interconnected Ni nanowire current collectors studied by Zankowski et al. have an ultra-high volume specific surface area of 26 m^2^/cm^3^ (Figure 8b), which solves the problem of low utilization of active substances in traditional bulk cathodes by uniformly distributing active substances [89]. This kind of grid structure promotes electrolyte penetration and ion diffusion by providing open channels while maintaining the continuity of the electron conduction path, achieving synergistic optimization of high rate performance and volume capacity.

Carbon nanotubes and graphene are ideal candidates for flexible cathodes due to their high specific surface area and flexibility. The flexible conductive skeleton reduces the crack formation of the cathode during the cycle through physical binding. Jo et al. promoted electrolyte penetration and lithium ion diffusion by replacing traditional metal current collectors with 3D carbon nanotube sheets (CNTS), forming an efficient electron transport network independent of metal substrates. Its excellent flexibility maintains excellent electrochemical performance under mechanical deformation (such as folding and bending) [62]. Similarly, the sandwich structure of the vertical channel and the graphene interlayer encapsulating the active material, constructed by the ice template method (Figure 8c), significantly reduces lithium ion diffusion tortuosity and improves ion transmission efficiency. It still maintains a capacity of 9.4 mA h/cm^2^ at a load of 72 mg/cm^2^, and the energy density is 739 Wh/L. Moreover, the ice template method does not require complex equipment, and the preparation cost of the “sandwich” cathode is lower than that of commercial carbon nanotube composites [37]. Peng et al. used a 3D-printed grid-structured cathode (Figure 8d), which exhibited minimal deformation and uniform stress distribution under unidirectional compression and multi-directional tensile loads, thereby addressing the mechanical failure of thick cathodes during packaging and charge–discharge cycles [92]. Kang et al. constructed a 3D electronic conduction network by intertwining binder-free carbon fibers (Figure 8e), physically intertwining carbon fibers with active particles to form a continuous conductive path throughout the cathode thickness [33]. Traditional slurry coating processes rely on carbon black conductive agents and polymer binders. In contrast, the carbon fiber network significantly reduces interfacial contact resistance. Additionally, it addresses the bottleneck of uneven electron conduction in high-load cathodes.

Inspired by natural wood materials with aligned channels along the tree growth direction, Chen et al. developed a multi-channel carbon framework as a 3D current collector. This framework features a conductive skeleton with low tortuosity (tortuosity ≈ 1.5) and high porosity (81%). These characteristics provide natural high-speed channels for ion and electron transport [88]. Through low-cost natural material derivatization, it breaks through the physical limitations of traditional cathode design and provides a general solution for high-load cathodes.

Through 3D reconstructing conductive networks in cathode microstructure, they address core issues in thick cathodes, such as limited electron/ion transport and insufficient mechanical strength. Consequently, these networks significantly enhance the energy density and power density of thick cathodes. This advancement provides a new idea for developing high-energy-density and high-power-density batteries.

#### 3.2.3. Cathode Architecture Optimization

**Figure 9:**
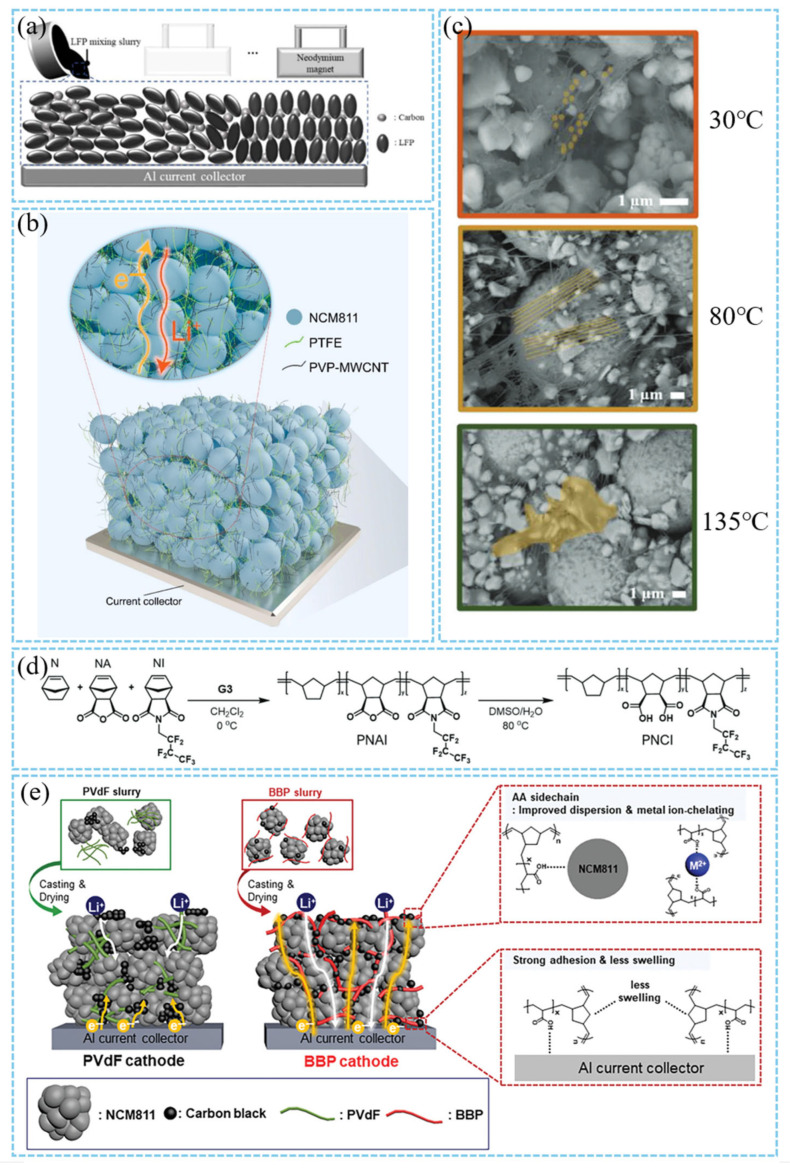
(**a**) Magnetic treatment schematic diagram of positive cathode slurry [95]. Reproduced with permission from [Springer Nature], [2022]. (**b**) Schematic illustration of the PVP encapsulating the surface of CNT through molecular chains [103]. Reproduced with permission from [John Wiley and Sons], [2025]. (**c**) SEM images of PTFE cathode composites after the fibrillation process at temperatures of 30 °C, 80 °C, 135 °C [100]. Reproduced with permission from [Royal Society of Chemistry], [2013]. (**d**) Synthesis of PNCI terpolymers [104]. Reproduced with permission from [Elsevier], [2025]. (**e**) Schematic representation depicting structural superiority of the BBP cathode over the PVDF cathode [105]. Reproduced with permission from [John Wiley and Sons], [2024].

The optimization of cathode architecture plays an important role in the design of thick plates, which directly affects the energy density, power performance, and cycle life of LIBs. By calendering, the cathode microstructure is fixed without further change. Modifying the cathode structure necessitates adhering to a four-step process: mixing, coating, drying, and calendering. Kim et al. mixed UV-curable gel electrolyte precursors in the cathode paste, avoiding the treatment solvents such as NMP used in the traditional cathode manufacturing process, thereby eliminating the solvent drying step, preventing the solvent-drying-triggered non-uniform distribution of cathode components, and shortening the battery aging time [93]. Chen et al. in situ coated an ultrathin red phosphorus nanolayer (≈2 nm) on the surface of commercial carbon black (CB) particles, which was converted into a stable Li3P nanolayer after the first activation of the battery. Li_3_P has high ionic conductivity (≈10^−3^ S cm^−1^), which significantly improves the Li^+^ migration ability inside the cathode. The Li^+^ transfer number (tLi^+^ = 0.67) is 60% higher than that of the traditional CB (tLi^+^ = 0.42), and the concentration polarization is reduced [94]. Kim et al. proposed a magnetically induced alignment strategy, in which LFP particles and carbon additives are aligned by an external magnetic field to form a uniformly distributed pore structure (Figure 9a), which significantly reduces the internal resistance of the cathode (the polarization is reduced by 6.3% at 1 C rate). This physical control method does not require chemical modification and avoids the risk of side reactions caused by traditional carbon coating [95]. Excessive porosity in thick cathodes will lead to increased electrolyte consumption and tortuous lithium ion diffusion paths. The pore structure can be optimized by adjusting the slurry composition and manufacturing process. Alolaywi et al. used severe calendering (compression ratio 43.5%) to prepare the NMC811 cathode with low porosity (6.1%), and formed a denser conductive network, which improved the continuity of the electron conduction path. The electronic resistance of the low porosity cathode decreased by 3~13 times, but severe rolling may lead to particle breakage [63]. Karanth et al. proposed an ethanol-induced phase transformation strategy. The surface of the phase-transformed cathode has a channelized macroporous structure with a pore size of 2.6 μm. In contrast, the traditional cathode has a small pore size of 1.8 μm. For the 35 mg/cm^2^ cathode, the capacity reaches 131.7 mA h/g at 1 C. This capacity is 132% higher than that of the traditional cathode. [96]. In addition, other cathode manufacturing processes can also be used. Such as dry cathode manufacturing process, 3D printing cathode manufacturing [97], and so on. Dry cathode technology has become an important direction of thick cathode manufacturing because of avoiding component segregation and pore heterogeneity caused by solvent evaporation. The dry cathode technology simplifies the traditional “powder-slurry-film” process to the “powder-film” process and abandons the organic solvents (such as NMP) in the traditional wet process. As a solvent, NMP has toxicity, high cost, and environmental pollution problems. The recovery and drying of NMP account for 78% of the cathode production cost, and its volatility poses a threat to workers’ health [38]. In the dry cathode manufacturing process (PVDF/CB = 1:1), the cathode has both high mechanical strength (peel strength of 162.77 N/m) and excellent electron/ion conductivity (ionic conductivity of 1.024 mS/cm), which is significantly better than the traditional wet cathode [41]. The high PVDF/CB ratio leads to an increase in the ion blocking effect and interfacial resistance, while the low PVDF/CB ratio optimizes the conductive network and Li^+^ diffusion kinetics. PTFE fibrous binder can replace PVDF to realize dry cathode manufacturing. At 80 °C, uniform fibrillation of PTFE is achieved (Figure 9c), significantly improving the dispersion of binder and carbon black (CB), making the pore distribution uniform, and reducing the risk of cathode pore blockage. The Li^+^ diffusion channel is formed through the fiber network to reduce the tortuosity factor. The point contact bonding reduces the conductive agent coverage area, exposes more active sites, and reduces the charge transfer resistance. However, in comparison to conventional binders (e.g., PVDF), PTFE exhibits a significantly higher material cost [98,99,100,101]. Carbon nanotubes are often used to construct excellent conductive networks due to their high aspect ratio and excellent conductivity. However, when they are mixed without solvents, their agglomeration tendency limits their contact with active substances. Kim et al. used gas-phase ozone oxidation for surface modification of single-walled carbon nanotubes (SWCNTs) to replace traditional liquid-phase strong acidic oxidizers [85]. The electrochemical performance of the dry cathode was significantly improved. Compared with traditional carbon black (CB), the O-SWCNT cathode exhibits higher initial coulombic efficiency (92.8% vs. 90.5%), better cycle stability (capacity retention after 100 cycles, 85.6% vs. 57.0%), and rate performance [102]. C.N. et al. combined polyvinylpyrrolidone (PVP) with ethanol to construct a non-solvent dispersion system. PVP encapsulates the surface of CNT through molecular chains to form a stable dispersion (Figure 9b). The low boiling point of ethanol accelerates the volatilization of the solvent after mixing, retains the integrity of the cathode components, and effectively solves the agglomeration problem of carbon nanotubes (CNT) in the preparation of dry cathodes [103].

The traditional PVDF binder leads to poor interface stability and is difficult to adapt to the mechanical stress of thick cathodes. The amorphous PVDF (crystallinity 45.7%) obtained by quenching process can significantly improve the electrolyte wettability (liquid absorption rate 25.8%) and ion-conducting lithium capacity (charge transfer resistance *R*_ct_ = 68.5 Ω) [106]. The new binder takes into account mechanical strength and interface compatibility through molecular design. By introducing polar carboxylic acid and nonpolar perfluoroalkyl moieties, the PNCI series copolymer binders developed by Jeong et al. realized the dual effects on NCM811 particles. On one hand, the interaction with NCM surface and Al current collector was enhanced by hydrogen bonding. On the other hand, the fluorine element was used to reduce the surface energy, prevent the agglomeration of NCM and conductive carbon, and promote electrolyte infiltration (Figure 9d) [104]. Kim et al. proposed amphiphilic bottlebrush polymers (BBP) that combine a hydrophilic poly (acrylic acid) (PAA) side chain with a hydrophobic polynorbornene (PNB) main chain (Figure 9e). These polymers maintain a low electrolyte swelling rate. Additionally, TM ion dissolution is inhibited by chelation. Consequently, the NCM811 cathode exhibits a stable cycle retention rate under a high load of 27 mg/cm^2^ [105]. A fluorine-free and hydroxyl-rich siloxane nanohybrid (SNH) binder reported by Jang et al. has a lower storage modulus (17.92 Pa vs. 42.55 Pa of PVDF). The SNH slurry had higher shear-thinning behavior and coated the cathode more uniformly than the PVDF slurry, which was more conducive to the preparation of high-load cathode. In the half-cell, the capacity retention of the SNH cathode is 81.9% after 200 cycles, which is significantly better than that of the PVDF cathode (58.8%) [106].

In summary, the manufacturing process of the cathode is crucial to the structure of the cathode. Future research needs to further explore new intelligent manufacturing technologies (such as 4D printing) investigate low-cost green synthesis paths, develop low-temperature activated binders or non-PVDF systems to reduce energy consumption and cost, and promote the large-scale application of thick cathodes in high-energy-density scenarios (e.g., electric vehicles).

### 3.3. Electrode/Electrolyte Interfacial Design

Electrode/electrolyte interface design plays an important role in the design of thick plates, which directly affects the energy density, cycle stability, and safety of the battery. There is a linear relationship between the interface resistance and capacity decay of thick cathodes. The key to improve the electrochemical performance of thick cathodes is to reduce the interface impedance [107]. Recent studies have shown that optimizing the electrode/electrolyte interface through interface engineering, inhibiting interfacial side reactions, and enhancing structural stability are effective strategies to improve the performance of thick cathodes.

**Figure 10:**
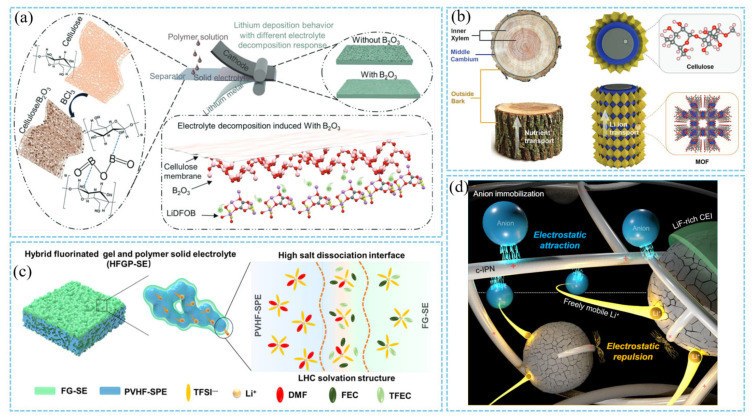
(**a**) Schematic illustration of Li deposition in the pouch cell assembled with cellulose/B_2_O_3_-based solid electrolyte [113]. Reproduced with permission from [John Wiley and Sons], [2023]. (**b**) The Li-MOF (metal–organic framework)/cellulose structural diagrams with the tree-trunk structure [40]. Reproduced with permission from [John Wiley and Sons], [2022]. (**c**) Designed a hybrid fluorinated gel and polymer solid electrolyte [39]. Reproduced with permission from [Royal Society of Chemistry], [2025]. (**d**) Schematic diagram of the regulation of electrostatic phenomena by cationic polymer binders [114].

First of all, the interface stability between electrolyte and electrode is the key to the long-term cycle of thick cathode, which plays a decisive role in energy density and cycle life. For high nickel cathodes (such as NCM811), the decomposition of electrolyte leads to the dissolution of transition metal and the increase in interface impedance [108]. Ether electrolytes have attracted much attention due to its higher lowest unoccupied molecular orbital energy level and more stable solid electrolyte interface (SEI) [109,110]. For example, Zhang et al. can form a stable cathode–electrolyte interphase (CEI) layer rich in LiF and Li_2_CO_3_ on the cathode surface by introducing fluorine-containing solvents (e.g., fluoroethylene carbonate, FEC) or additives (e.g., LiDFOB) (Figure 10c). This CEI layer effectively inhibits side reactions and enhances oxidation stability. However, the insufficient oxidation stability (usually lower than 4.0 V) of the ether electrolyte limits its compatibility with high nickel cathodes (such as NMC622 and NMC811). Dato et al. used fluorobenzene (FB) as a cosolvent and introduced a high-voltage organic sulfur electrolyte system, breaking through the limitations of traditional fluorinated ether diluents (such as TPE, TTPE, and TTEE). FB does not contain a fluorinated ether structure, and its unique electronic effect gives it excellent chemical stability [111]. By reducing electrolyte viscosity, enhancing lithium-ion migration, and inhibiting side reactions caused by reactive oxygen species, the long cycle life and high rate performance of NCM811 can be realized. Yang et al. constructed a dense CEI film on the surface of NCM811 through the synergistic effect of LiTFSI (providing Li^+^ conduction), LiDFOB (regulating SEI component), and LiBF_4_ (improving oxidation stability), so that the battery can maintain 83% capacity retention after 100 cycles at a high voltage of 4.5 V [112].

Secondly, the interface compatibility between the electrolyte and the lithium metal anode directly affects the dendrite suppression and cycle life. The traditional liquid electrolyte is prone to side reactions with lithium, resulting in the instability of the SEI film. Cheng et al. designed a cellulose-based composite solid electrolyte modified with boron oxide (B_2_O_3_) solid acid (Figure 10a). This electrolyte induced the formation of a LiF/Li_2_CO_3_ heterogeneous-structured SEI layer. It also inhibited lithium dendrite growth and reduced interface impedance. These effects significantly improved the uniformity of lithium deposition. Consequently, the symmetric battery could be stably cycled for over 200 h at a current density of 0.5 mA/cm^2^. In contrast, the cellulose-based cell short-circuited after only 24 h [113]. In addition, the trunk structure design proposed by Zheng et al. optimized the transmission path of lithium ions through layered ion channels, and used cellulose as the inner frame (similar to the xylem of trees) to provide mechanical support; the metal–organic framework (MOF) layer (similar to the bark of trees) is grown in situ on the surface of cellulose to form an ion transport channel and a protective layer (Figure 10b), reducing concentration polarization. As a result, the thick cathode battery still maintains 80% capacity after 3000 cycles [40].

For the liquid electrolyte system, an ultra-thin Li_3_PO_4_ layer deposited on the LiNi_0.5_Mn_1.5_O_4_ surface by ALD technology can form a uniform ionic conductive interface, which significantly reduces electrolyte decomposition and manganese dissolution. The unprotected 7400 nm thick LNMO film has a capacity retention rate of only 66% after 34 cycles at C/2 rate. After the deposition of 3 nm thick Li_3_PO_4_ layer by ALD, the capacity retention rate is increased to 92%, and the cycle life is extended by 230 times. However, the LNMO film will still undergo mechanical degradation during the long-term cycle [115]. In addition, the cationic polymer binder maintains the dispersion of the electrode particles through electrostatic repulsion and reduces the generation of cracks during the cycle (Figure 10d), thereby improving the structural integrity of the high area capacity electrode [114]. This interface regulation strategy not only optimizes the Li transport kinetics but also enhances the mechanical stability of the electrode and provides a new idea for the design of thick cathodes.

In terms of electrolyte materials, solid-state electrolytes (SSEs) are considered to be a promising solution to the dilemma between high energy density and safety due to their non-flammability and ability to inhibit the growth of lithium dendrites. Therefore, the construction of all-solid-state lithium metal batteries by replacing flammable liquid electrolytes with solid electrolytes is considered to be a promising solution to the dilemma between high energy density and safety [116,117]. The research of solid-state lithium metal batteries mainly focuses on improving the ionic conductivity of the solid electrolyte and optimizing the cathode structure. Dong, Liang et al. developed a new type of single-ion conductor electrolyte to address the challenges of thick cathode sheets. This electrolyte achieves high Li^+^ conductivity (>0.4 mS/cm at 20 °C) and a lithium-ion transference number (tLi^+^ = 0.96). It also exhibits a wide electrochemical window (>4.8 V) and compatibility with high-voltage NMC811 cathodes. After cycling, no dendrite formation is observed on the lithium electrode surface, and the interface between the electrolyte and electrode remains intact [118,119]. He et al. constructed a solid electrolyte with a wide temperature range (−20~60 °C) and stable cycle by in situ polymerization of polyethylene glycol (PEG)-based monomer and low melting point solvent (1,3-dioxolane or ethyl difluoroacetate). The capacity retention rate was 81% after 1300 cycles at 5 C under room temperature, and it remained 80% after 500 cycles at 60 °C under high-temperature conditions [120].

In sulfide all-solid-state lithium batteries, the interface instability between nickel-based ternary cathode materials (such as NCMA) and sulfide electrolytes is a key factor limiting their cycle life. By constructing a core–shell structure on the surface of NCMA and combining with LiNbO_3_ coating, Yang et al. can effectively inhibit the interfacial side reactions and alleviate the structural degradation caused by phase transition, so that the capacity retention rate is 96.4% after 300 cycles at 0.5 C under the high loading of 35.6 mg/cm^2^, which is significantly better than that of the uncoated sample (87.3%) [121]. Similarly, the mechanical fusion coating of LiZr_2_(PO_4_)_3_ nano-particles can not only optimize the Li diffusion channel but also stabilize the cathode structure through Zr doping, and maintain a high-capacity retention rate under 54 mg/cm^2^ loading [122]. These studies have shown that interfacial modification can improve the cycle reliability of thick cathodes through the dual mechanisms of physical isolation and chemical stability.

In addition, the thickness of the solid electrolyte has a direct effect on the interface contact and ion transport efficiency. The ultra-thin (≈7 μm) multifunctional polymer electrolyte (only 1/50 of the commercial separator) proposed by Zhang et al. significantly shortens the lithium ion diffusion distance and reduces the ohmic polarization [123]. Its ionic conductivity at room temperature reaches 5.3 × 10^−5^ S/cm, which supports the soft pack battery to light up the LED after 70 cycles at 0.5 C [123]. It is worth noting that the interface design needs to take into account the material selection and process suitability. For example, the small-sized single-crystal nickel-based cathode combined with pre-lithiation treatment can maintain structural stability at high voltage [124]. Additionally, the synergistic effect of B doping and coating further enhances the cathode’s oxidation resistance [125]. These multi-dimensional interface optimization strategies show that a single modification method is often difficult to meet the needs of complex interfaces, and performance breakthroughs need to be achieved through the combination of material innovation and interface engineering.

Interface design plays a central role in the design of thick cathodes. It regulates the electrode/electrolyte interface behavior, optimizes the ion transport path, and enhances the structural stability. These combined effects serve as a key breakthrough for realizing the practical application of high-energy-density thick cathode batteries.

### 3.4. Other Strategies

In addition to the common thick cathode performance optimization strategies, Plateau et al. proposed a micro-casting technology based on micron-scale patterned scrapers [126]. The pore distribution inside the cathode was optimized by alternating density structure (SDP) design, which significantly reduced the porosity (from 35% of the traditional cathode to 15%) while maintaining a high cathode compaction density (>3.5 g/cm^3^). The SDP structure shortens the diffusion distance of lithium ions, and the diffusion coefficient is three times higher than that of the traditional cathode, which alleviates the polarization problem of the thick cathode, inhibits the structural collapse during the cathode drying process, and reduces the shedding of the active material [126]. By introducing permanent magnets (such as NdFeB) into the battery, Chen et al. used Lorentz force to induce directional migration of lithium ions, homogenize local current density, and inhibit dendrite growth without modifying cathode materials or electrolytes [127]. Experiments show that the magnetic field can increase the first-cycle coulombic efficiency of the anode-free lithium metal battery from 70% to 98%, and the capacity retention rate is increased by 30% after 200 cycles [127]. The micro-casting process significantly increases the volume energy density through pore structure optimization, and magnetic field regulation provides a new control dimension for ion diffusion and metal deposition [127]. Such designs provide innovative inspiration for the development of high-performance thick cathodes.

## 4. Summary and Outlook

**Figure 11:**
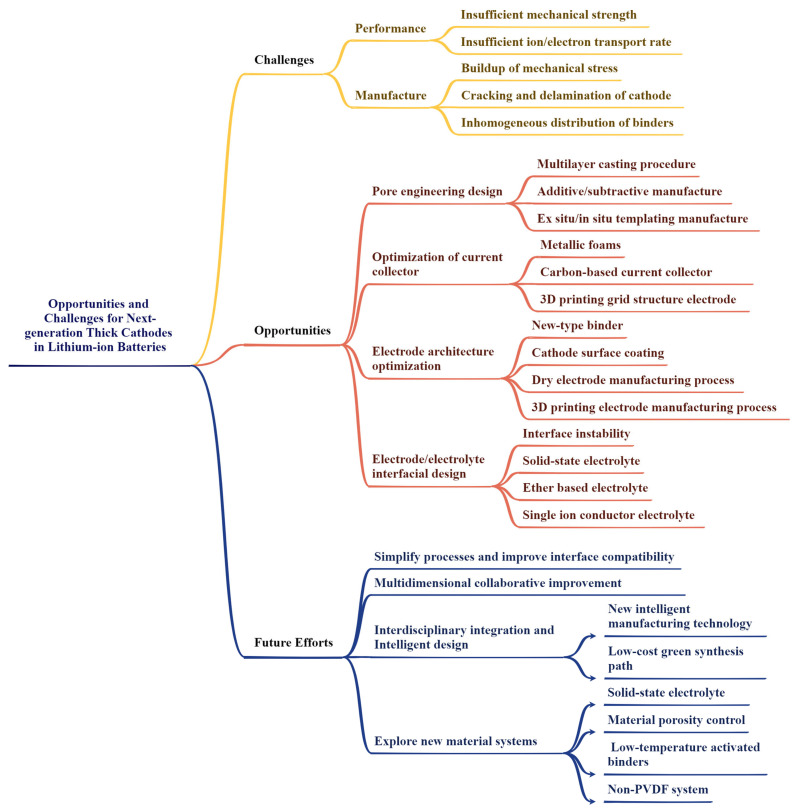
Schematic diagram of the challenges and future improvement strategies of the thick cathode design.

In summary, compared with traditional cathodes, thick cathodes have the potential to increase energy density of LIBs due to their high active material loading, but there are also problems, such as insufficient mechanical strength and lithium-ion transport rate. Considering the thick cathodes design acting as the key technology to improve the energy density of the LIBs, the future development needs to make breakthroughs in structural design, material innovation, manufacturing process, and large-scale application (Figure 11). A detailed comparison of different thick cathodes was summarized in Table 1. regarding their various modification strategies and corresponding electrochemical performances systematically. Although there are various solutions to solve the problems related to the performance and manufacturing of thick cathodes, significant progress is still needed to achieve efficient manufacturing on the scale of modern factories. The microstructure of the cathode is fixed by the calendering step, which determines the electrochemical performance of the cathode. According to the cathode material and the reaction mechanism, the appropriate cathode structure design should be selected to achieve the construction of the microstructure and the maximization of the electrochemical performance. This paper discusses the barriers to current thick cathodes modification technologies and proposes potential opportunities to develop stronger and thick cathodes while ensuring long-term performance and scalability.

Insufficient mechanical strength will lead to the formation of cracks on the cathode surface and finally result in cathode stripping. The slow charge transfer of thick cathodes will also lead to deterioration of reaction kinetics. Single-crystal particles have stronger mechanical strength than polycrystalline particles. Through gradient design, surface coating modification, manufacturing process optimization, etc., the mechanical strength can be well improved. The insufficient electrochemical performance of the thick cathode is mainly related to the insufficient ion and electron transport. Ion transport is usually related to pore structure which can be enhanced by constructing vertically aligned layered channels, such as ex situ/in situ templating manufacture, additive/subtractive manufacture, multilayer casting procedure, and 3D printing. The transport of electrons is mainly related to the conductive network, including the type and morphology of conductive agent, conductive network structure, porosity, and so on. By introducing multi-dimensional conductive materials for synergistic effects (carbon black, CNTs, graphene, etc.), the interface resistance can be significantly reduced. Adjusting the particle size of the cathode material, along with the distribution and type of binder, can enhance electron transport. Many challenges in thick cathodes lie in identifying a suitable range of process parameters. For example, optimizing porosity is an equilibrium process between electron and ion transport. Increasing porosity helps reduce bending and enhances ion transport, but excessive porosity disrupts the electron conduction path, whereas reducing porosity increases the proportion of active materials.

In the future, it is necessary to adopt several modification strategies simultaneously among process optimization, material innovation, and technological breakthroughs with the aim to promote the large-scale application of thick cathodes in commercial LIBs. First of all, it is necessary to further simplify the preparation process to improve the cathode interface compatibility and explore new material systems actively. In this process, the precise control of material porosity, the optimization of interface characteristics and the development of large-scale preparation technology will serve as the key research directions. In addition, combined with the upgrading of intelligent manufacturing technology, the capital cost of thick cathodes in LIBs can be effectively reduced by the development of low-cost green synthesis paths, low-temperature activated binders and non-PVDF binder systems, which is beneficial to the commercial application of thick cathodes. Solid-state electrolyte is another approach to increase energy density. The integration of thick cathodes and solid-state electrolytes can further enhance the energy density of LIBs. By replacing liquid electrolyte, the issue of uneven infiltration of liquid electrolyte in thick electrodes can be effectively resolved. Additionally, the ultra-thin solid-state electrolyte layer can further reduce the proportion of inactive materials and thus increase energy density. In the future, efforts should focus on four aspects: (i) simplifying the preparation process and improve interface compatibility; (ii) multidimensional collaborative modification strategies for the large-scale preparation of high-loading cathodes to promote the commercialization of 500 Wh/kg solid-state batteries; (iii) using interdisciplinary integration and intelligent design to develop new intelligent manufacturing technology and low-cost green synthesis path; and (iv) exploring new materials systems including new-type solid-state electrolyte, low-temperature activated binders, non-PVDF systems, and optimized material porosity.

By constructing a thick cathode, it not only improves the energy density of the battery but also provides new ideas for solving other key problems of the battery, such as inhibiting the growth of lithium dendrites and inhibiting interfacial side reactions. Prior to constructing thick cathodes, it is essential to fundamentally elucidate the relationship between cathode microstructure and electrochemical performance. The cathode microstructure is designed by adjusting the four steps of the cathode manufacturing process including mixing, coating, drying, and calendering. Such thick cathodes require fast ion and electron transfer rates, high mechanical strength, environmental protection, low cost and high scalability. Therefore, the future development of thick cathodes requires optimization in the preparation process regarding their porosity, tortuosity, and thickness simultaneously. It also necessitates implementing multi-collaborative modification strategies together to achieve simultaneous optimization both in performance and capital cost. This will provide crucial technical support for large-scale practical applications of high energy density batteries in the future.

## Figures and Tables

**Table 1:** Improvement strategies and electrochemical performance comparison of different thick cathode materials.

	Active Materials	Improvement Strategy	Cycling Stability	Electrode Capacity [mAh g^−1^/C]	Voltage Window [V vs. Li/Li^+^]	Loading [mg/cm^2^]	Scalability Level [1~5] ^(a)^	Ref.
Pore engineering	LiFePO_4_	Templated phase inversion	-	156/0.1 C	2.5–4 V	100	4	[35]
	Li(Ni_0.6_Mn_0.2_Co_0.2_)O_2_	Laser structure	72%/80 (0.5 C)	130/0.5 C	3–4.3 V	35	3.5	[50]
	LCO	Ice-template	90%/200 (1 C)	124/1 C	3–4.2 V	30~35	4	[54]
	LiNi_0.8_Mn_0.1_Co_0.1_O_2_	Multilayer coating process	-	171/0.2 C	2.8–4.5 V	20~25	4	[77]
	Ni-rich NCM	(Single crystal/polycrystalline) double layer cathode	55.5%/50 (0.5 C)	-	2.8–4.3 V	21	4.5	[79]
	LiNi_0.9_Co_0.05_Mn_0.05_O_2_	Gradient pore structure	88.24%/100 (1 C/2 C)	62.09/4 C	2.5–4.2 V	25	4.5	[80]
	LiNi_0.8_Mn_0.1_Co_0.1_O_2_	Adhesive gradient	93%/100 (0.2 C)	156/1 C	3–4.2 V	20~25	4	[81]
	LiNi_0.8_Co_0.15_Al_0.05_O_2_	Gradient porosity	99.5%/100 (0.2 C)	180.7/0.2 C	4.25 V	13~16	4	[85]
	LiNi_0.83_Mn_0.12_Co_0.05_O_2_	Particle size gradient	73.3%/150.05 mA h/g (1 C)	176.1/1 C	2.7–4.3 V	29.6	4.5	[34]
	NMC532	Particle size gradient	80%/1000 (0.5 C)	-	2.5–4.2 V	25	4	[84]
2D conductive percolation network	LiNi_1/3_Co_1/3_Mn_1/3_O_2_	2D porous nanosheets	92.8%/100 (0.1 C)	147.2/0.1	2.8–4.3 V	320	4	[59]
	LiNi_0.6_Co_0.2_Mn_0.2_O_2_	Single-walled carbon nanotubes	80%/300 (0.5 C)	4.7 mA h cm^−2^/0.5 C	2.5–4.3 V	36.4	4	[57]
	LiNi_0.8_Co_0.1_Mn_0.1_O_2_	Carbon nanofiber	93.7%/100 (1 C)	208.02/0.1 C	2.75–4.3 V	20	4	[86]
	LiMn_2_O_4_	Single-walled carbon nanotubes	95%/50 (0.1 C)	106/0.1 C	3–4.3 V	60	3	[58]
3D conductive scaffold	LiFePO_4_	Nickel alloy foam current collector	90%/100 (0.3 C)	102/0.3 C	2.5–4 V	32	4	[60]
	NCM811	CNTS prepared by spinning technology	89.6%/100 (2 C)	211/0.1 C	3–4.3 V	6.3	4	[62]
	LiFe_0.7_Mn_0.3_PO_4_	Vertical channel sandwich structure	60%/1000 (1 C)	146.8/0.5 C	2–4.5 V	21.2	4.5	[37]
	LiNi_0.8_Co_0.1_Mn_0.1_O_2_	3D printing grid structure	77.68%/100 (200 mA/g)	204.3/25 mA/g	2.8–4.3 V	36.6	4	[92]
	LiNi_0.6_Co_0.2_Mn_0.2_O_2_	3D carbon fiber network	84%/50 (1 C)	165/1 C	3–4.6 V	11	4	[33]
	LFP	Bionic multi-channel carbon framework	76%/140 (2 mA cm^−2^)	5 mA h cm^−2^/2 mA cm^−2^	2.15–4.2 V	60	4	[88]
Cathode architecture optimization	LiNi_0.8_Co_0.1_Mn_0.1_O_2_	Bicontinuous electron/ion conduction network	90%/80 (0.05 C)	191/0.05 C	3–4.2 V	36	4	[93]
	NMC811	Severe calendering process	87.7%/100 (0.33 C)	181/0.33 C	3–4.2 V	19.13	4	[63]
	NMC811	Non-solvent induced phase transformation technology	98.65%/100 (0.1 C)	160.3/1 C	3.6–4.3 V	60	4.5	[96]
	LFP	3D printing technology	-	133/0.2 mA cm^−2^	-	108	4	[97]
	LiNi_0.8_Co_0.15_Al_0.05_O_2_	Roll-to-roll drying process	82.1%/100 (0.5 C)	190.1/0.5 C	-	50	4	[100]
	NCM811	PTFE Adhesive/Dry Process	-	160/0.5 C	-	52	4.5	[99]
	LiNi_0.8_Co_0.1_Mn_0.1_O_2_	Carbon nanotube dispersion/dry process	66.1%/50 (0.33 C)	211.47/0.2 C	2.8~4.4 V	50	4	[103]
	LiNi_0.8_Mn_0.1_Co_0.1_O_2_	Adhesive optimization	75%/300 (0.5 C)	-	3.0–4.5 V	21.7	4.5	[104]
	LiNi_0.8_Mn_0.1_Co_0.1_O_2_	Design of new adhesive	80.6%/240 (0.5 C)	190/0.1 C	3.0–4.2 V	27	4	[105]
Electrode/electrolyte interfacial design	LiNi_0.8_Mn_0.1_Co_0.1_O_2_	Nonsolvating fluoroaromatic cosolvent	71.9%/500 (0.33 C)	218.9/0.33 C	3.0–4.5 V	13.75	4	[111]
	NMC622	Concentrated ternary salt ether-based electrolyte	80%/430 (0.2 C/0.5 C)	-	2.7–4.4 V	13.8	4	[112]
	NCM811	“Tree-Trunk” design	80%/300 (1 C)	207/0.1 C	-	14.8	4	[40]
	LiNi_0.8_Co_0.1_Mn_0.1_O_2_	Cationic polymer binder	82%/100 (0.68/1.35 mA cm^−2^)	210/0.1 C	3.0–4.4 V	65	4	[114]
	NCM811	Single-ion conductor polymer electrolyte	84.5%/300 (2 C)	160/0.3 C	3.0–4.2 V	10.6	4	[118]
	LiFePO_4_	Novel quasi-solid polymer electrolyte	75%/1500 (2 C)	116/2 C	-	6~7	4.5	[120]
	LiNi_0.88_Co_0.04_Mn_0.05_Al_0.03_O_2_	Core–shell structure engineering and surface coating synergy	96.4%/300 (0.5 C)	128.8/2 C (55 °C)	2.1–3.68 V	35.6	4	[121]
	LiNi_0.8_Mn_0.1_Co_0.1_O_2_	Mechanical melting modification	97%/1300 (1 C)	194.9/0.05 C	2.8–4.4 V	27	4	[122]
	NCM811	Ultrathin multifunctional polymer electrolyte	84.2%/500 (0.5 C)	178.6/0.5 C	2.8~4.3 V	7~9	4.2	[123]

(a) The ranking is divided into 5 levels based on the potential for technological scalability. The highest 5th level represents the highest scalability for technological industrialization.

## Data Availability

No new data were created or analyzed in this study. Data sharing is not applicable to this article.
